# Environmental Enrichment Preserves Retrosplenial Parvalbumin Density and Cognitive Function in Female 5xFAD Mice

**DOI:** 10.1523/JNEUROSCI.0455-25.2026

**Published:** 2026-02-03

**Authors:** Dylan J. Terstege, Jonathan R. Epp

**Affiliations:** Department of Cell Biology and Anatomy, Hotchkiss Brain Institute, Cumming School of Medicine, University of Calgary, Calgary, Alberta T2N 4N1, Canada

**Keywords:** Alzheimer's disease, enriched housing, perineuronal nets, retrosplenial cortex

## Abstract

The rate of cognitive decline in Alzheimer's disease (AD) varies considerably from person to person. Numerous epidemiological studies point to the protective effects of cognitive, social, and physical enrichment as potential mediators of cognitive decline in AD; however, there is much debate as to the mechanism underlying these protective effects. The retrosplenial cortex (RSC) is one of the earliest brain regions with impaired functions during AD pathogenesis, and its activity is affected by cognitive, social, and physical stimulation, making it a particularly interesting region to investigate the influences of an enriched lifestyle on AD pathogenesis. In the current study, we use the 5xFAD mouse mode of AD to examine the impact of enriched housing conditions on cognitive function in AD and the viability of a particularly vulnerable cell population within the RSC—parvalbumin interneurons (PV-INs). Enriched housing conditions improved cognitive performance in female 5xFAD mice. These changes in cognitive performance coincided with restored functional connectivity of the RSC and preserved PV-IN density within this region. Along with preserved PV-IN density, there was an increase in the density of *Wisteria floribunda* agglutinin-positive perineuronal nets (WFA^+^ PNNs) across the RSC of 5xFAD mice housed in enriched conditions. Direct manipulation of WFA^+^ PNNs revealed that these extracellular matrix structures protect PV-INs from amyloid toxicity and may be the mechanisms underlying the protective effects of enrichment. Together, these results provide support for the WFA^+^ PNN-mediated maintenance of PV-INs in the RSC as a potential mechanism mediating the protective effects of enrichment against cognitive decline in AD.

## Significance Statement

The rate of progression of Alzheimer's disease is highly variable. The extent to which individuals engage in an enriched lifestyle is one factor that has been proposed to promote cognitive resiliency to AD pathology. Understanding how enrichment promotes resiliency is critical for promoting healthy cognitive aging. Recent work has demonstrated that the retrosplenial cortex (RSC) and especially parvalbumin interneurons (PV-INs) in this region are highly vulnerable to AD pathology, and their impairments relate to early cognitive impairments. Here, we show that environmental enrichment promotes cognitive performance and the survival of PV-INs in the RSC through a mechanism dependent on perineuronal net maintenance. These results help to explain the mechanisms that mediate the influence of environmental enrichment on cognitive resiliency.

## Introduction

Alzheimer's disease (AD) is a neurodegenerative disorder characterized by progressive cognitive decline. Along with cognitive changes, AD is associated with pathological changes including the accumulation of amyloid plaques and neurofibrillary tangles (DeTure and Dickson, 2019). However, changes in brain function can be detected even prior to the development of these particular pathological signs (Frisoni et al., 2017; Georgakas et al., 2023). Furthermore, the degree of amyloid plaque and neurofibrillary tangle accumulation are relatively poor predictors of cognitive decline (Katzman et al., 1988; Ansart et al., 2021; Dansson et al., 2021). One factor that complicates the predictive power of these hallmark pathological signs is environmental enrichment. Individuals with a high degree of cognitive, social, and physical stimulation show an increased resilience to cognitive decline even when faced with similar pathological burden (Stern, 2012; Mondini et al., 2016; van Loenhoud et al., 2019).

Numerous epidemiological studies have identified protective effects of cognitive, social, and physical enrichment against disease progression in AD (Milgram et al., 2006; Hsiao et al., 2018; Meng et al., 2020). However, the underlying mechanisms which promote this cognitive resilience are not yet fully understood. Many studies have focused on isolating the individual influences of cognitive, social, and physical enrichment on resilience to cognitive decline. While this has great value in identifying potential specific mechanisms which may underlie the protective effects of each specific intervention, it has been demonstrated that these manipulations are most effective in tandem (Santoso et al., 2020). This combined enrichment likely gives rise to a wide variety of enhancements throughout the brain. In the current study, we have focused on the interplay between enrichment and retrosplenial cortex (RSC) dysfunction, an early predictor of the transition between MCI and AD (Terstege et al., 2024).

One of the earliest functional changes that can be detected in the brains of individuals prior to the onset of AD is dysfunction within the RSC (Ash et al., 2016; Dillen et al., 2016; Trask and Fournier, 2022). Hypometabolism in the RSC can be detected years before AD diagnosis (Minoshima et al., 1997; Nestor et al., 2003b) and, the severity of RSC hypometabolism may be predictive of transition from MCI to AD (Huang et al., 2002; Chételat et al., 2003; Nestor et al., 2003a; Terstege et al., 2024). As fast-spiking cell populations, parvalbumin-expressing interneurons (PV-INs) have high metabolic demands and are highly vulnerable to hypometabolism. As the most abundant source of GABAergic signaling in the RSC, PV-INs are critical for maintaining the excitatory–inhibitory balance of this region and are thus crucial for many higher-order cognitive processes. Several reports have identified impaired activity and expression of PV-INs in AD, particularly in the RSC (Hof et al., 1991; Bernstein et al., 2011; Terstege and Epp, 2023; van Heusden et al., 2023). Impaired PV-IN activity in the RSC arises earliest during AD pathogenesis in females, with this earlier susceptibility having been proposed as a causal factor influencing sex differences in AD vulnerability (Terstege et al., 2025).

Here, we examined the effects of long-term multimodal enrichment on cognitive performance and RSC function in female mice. We demonstrate impaired cognitive function in 5xFAD mice housed under standard housing conditions, but not in 5xFAD mice housed in enriched environments. Underlying impaired cognitive performance, 5xFAD mice housed under standard conditions displayed disrupted functional connectivity of the RSC and impaired expression of PV-INs. These impairments were not observed in 5xFAD mice housed in enriched environments. Furthermore, the density of *Wisteria floribunda* agglutinin-positive perineuronal nets (WFA^+^ PNNs) in the RSC was impaired in 5xFAD mice housed under standard conditions, with remaining PNN expression being more closely restricted to PV-INs. Finally, when WFA^+^ PNNs were depleted from the RSC of 5xFAD mice housed under enriched conditions, the protective effects on PV-IN density were no longer present. Together, these results suggest WFA^+^ PNN-mediated maintenance of RSC PV-INs as a potential mechanism through which enrichment promotes resilience to cognitive decline in AD.

## Materials and Methods

### Mice

Female mice were used for all experiments. For enrichment experiments, heterozygous 5xFAD mice were produced via in vitro fertilization by the University of Calgary Centre for Genome Engineering. Sperm was obtained from male homozygous 5xFAD mice (#034840-JAX), and female C57Bl/6J mice were used as oocyte donors (#000664-JAX). Mice were 8 weeks old at the start of the experiment, and all procedures were completed by the time these mice were 6 months old (24 weeks). All histological analyses in 5xFAD mice were conducted at 6 months old, while behavioral testing occurred at 4 months old (Y maze spontaneous alternation) and 6 months old (Y maze spontaneous alternation and trace conditioning).

For amyloid and chondroitinase infusion experiments, homozygous female C57Bl/6J mice were purchased from the Jackson Laboratory. In this series of experiments, 8-week-old mice were randomly assigned to treatment groups and received acute infusions. These mice were perfused 7 d later for histological analyses.

For experiments examining the combined influence of chondroitinase and enrichment, male 5xFAD mice were crossed with female PVcre mice (#017320-JAX). In PVcre mice, PV-INs express Cre recombinase without disrupting the endogenous expression of *Pvalb* (Hippenmeyer et al., 2005). Using this system, viral constructs containing loxP sites can be used to drive the expression of a gene of interest, specifically within PV-INs. Only 5xFAD+/PVcre+ and 5xFAD-(WT)/PVcre+ mice were used for these experiments. These mice were 4 months old at the start of the experiment, and all behavioral and histological procedures occurred 2 months later when mice were 6 months old. Throughout all experiments, the room lighting in housing facilities was maintained on a 12/12 h light/dark cycle (8 A.M., lights on). All procedures were conducted during the light cycle phase. All experiments involving animal subjects were conducted in accordance with Canadian Council on Animal Care guidelines and with the approval of the University of Calgary Animal Care Committee.

### Human tissue samples

The frozen-fixed RSC tissue from female patients with AD (mean age ± SD; 72.7 ± 2.6) and age-matched individuals without AD (mean age ± SD; 71.6 ± 3.0) was obtained from Douglas-Bell Canada Brain Bank. The known average duration of disease in these patients was 7.4 ± 4.6 years. Cognitively normal (CN) control and AD designations were defined with the support of medical charts and coroner records, in collaboration with the Quebec Coroner's Office. All patient information and tissue samples were obtained with informed consent from the next of kin. Toxicological assessments, medication prescriptions, and cognitive assessments were also obtained. All experiments involving human tissue samples were conducted in accordance with the University of Calgary Conjoint Health Research Ethics Board (REB22-0776).

### Enrichment

Custom enrichment cages, based on a previous design (Fares et al., 2013), were constructed to provide continuous enrichment. These large cages measured 61 × 40.5 × 37.5 cm (length × width × height) and contained a divider which separated a compartment containing food from a second compartment containing water. To pass from one compartment to the other, mice had to climb up a ladder to the second level of these cages and pass through an interchangeable maze insert. Maze inserts served as cognitive enrichment and were changed every 3 d. Six maze inserts were rotated through each cage and, over time, additional removable walls were added within these mazes to further modify their design. Each maze configuration was used only once, and the configurations of these maze inserts were identical for each enrichment chamber. In addition to interchangeable maze inserts, mice in the enriched condition were also given access to running wheels for physical enrichment. Finally, with larger cages, mice received social enrichment via an increased number of cage mates (8–12 mice per cage). Mice randomly assigned to the control conditions were housed in standard cages with 4–5 mice per cage and *ad libitum* access to food and water.

### Behavioral testing

Mice were habituated to handling for 5 d prior to all behavioral tasks. Mouse behavior was tracked using ANYmaze Behavioural Tracking Software (Stoelting).

#### Y maze spontaneous alternation

Spatial working memory ability was assessed using a Y maze spontaneous alternation task (Kraeuter et al., 2019; Khan et al., 2024). The arms of the Y maze apparatus were 41.5 cm long, 7.5 cm wide, and 12.5 cm tall and were cleaned with 70% EtOH before and after each trial. Mice were placed at the end of one arm of the Y maze allowed 5 min to explore the apparatus. During this trial, a spontaneous alternation was defined as a sequence in which mice entered each of the three arms of the maze without re-entering either of the two previously entered arms. The number of spontaneous alternations was expressed as a percentage of the total number of arm entries, with the first two arm entries excluded, for statistical comparisons between groups.

#### Trace conditioning

A trace fear conditioning protocol was used to assess the acquisition and recall of contextually conditioned and cue-associated memories (Kohman et al., 2012; Seo et al., 2015). Trace conditioning took place in Ugo Basile contextual fear conditioning chambers (17 cm wide × 17 cm wide × 25 cm high) placed inside of sound-attenuating cabinets. The conditioning chamber had black and white vertical stripes along the walls and metal bars along the floor and was cleaned with 30% isopropyl alcohol before and after each trial. During a conditioning trial, mice were allowed to explore the chamber for 3 min prior to the onset of a 20 s tone (2,700 Hz). A 20 s trace period followed the offset of this tone prior to the delivery of a footshock (1 mA, 2 s). The tone-trace-shock presentation was repeated four times (five presentations in total) with a 200 s intertrial interval. Mice were removed from the conditioning chamber 1 min after the final shock and returned to the home cage.

Trace memory testing took place 24 h after the conditioning trial in a distinct context. These chambers had solid gray walls and floors and an open vial of vanilla extract underneath the floor. Chambers were cleaned with 70% EtOH before and after each trial. During this trial, mice were allowed to explore the chamber for 2 min prior to the onset of a 20 s tone. Tones were presented with an intertrial interval of 220 s, with a total of three tones having been presented. During subsequent analyses, the 20 s following the offset of the tone was considered to be the trace period.

Contextual memory testing took place 48 h after the conditioning trial, during which mice were returned to the conditioned context for an 8 min trial without any shocks or tones. In both testing sessions, freezing was used as the primary measure of memory retention and was defined as a complete lack of motion, except for respiration, for at least 1 s.

### Surgical procedures

Surgeries were conducted under isoflurane anesthesia delivered via a SomnoSuite anesthetic delivery system (Kent Scientific). Mice were induced at 5% isoflurane, transferred to a stereotaxic frame, and maintained at 1–2% isoflurane. Mice were administered analgesia (Metacam, 5 mg/kg) and fluid support (warmed saline, 0.5 ml) at the beginning of the procedure and monitored closely throughout. A robotic stereotaxic manipulator paired with a stereodrive software (Neurostar) was used to drill burr holes and guide a glass infusion needle into place. Infusions were administered using a Nanoject III infusion system (Drummond Scientific) using the injection parameters and solutions described in the following, experiment-specific, sections. After each series of injections, the pipette was left at the target depth for 5 min following the final pulse to allow for diffusion of the solution. Once all injections were delivered, the incision was closed with suture material, and mice were removed from the stereotaxic frame and were monitored for 1 week during their recovery.

#### Acute chondroitinase and amyloid infusions

All mice received an injection of chondroitinase (ChABC, prepared at 100 units/ml in saline; Sigma-Aldrich, C3667-5UN) at an RSC target in the right hemisphere (AP, −2.2; ML, 0.5; DV, 1.0) and a saline injection at an RSC target in the left hemisphere (AP, −2.2; ML, −0.5; DV, 1.0). ChABC is an enzyme that disassembles PNNs by selectively cleaving chondroitin sulfate- and dermatan sulfate-glycosaminoglycan into disaccharide units (Miyata and Kitagawa, 2015; Alonge et al., 2020). At each target, these injections were delivered in five sets of 100 nl pulses at a rate of 10 nl/s with 10 s between pulses. Half of the mice (*n* = 5) then received bilateral injections of amyloid-β 1–42 (prepared at 1 mg/ml and incubated for 3 d at 36°C prior to use; Tocris Bioscience, 1428; 100 nl/site), while the other half (*n* = 5) received bilateral saline injections (100 nl/site).

To assess the potential influence of impurities in the ChABC formulations, an additional cohort of mice received bilateral injections of heat-inactivated ChABC (AP, −2.2; ML, ±0.5; DV, 1.0). The ChABC enzyme was heat-inactivated via incubation at 85°C for 45 min, a protocol which has previously been validated with in vitro digestion assays (Alonge et al., 2020).

#### Chronic chondroitinase and enrichment

Mice were randomly assigned to receive injections of AAV1-hSyn-DIO-ChABC (Carstens et al., 2021; 5xFAD, *n* = 4; WT, *n* = 5) or pAAV1-CAG-FLEX-tdTomato (Addgene #28306-AAV1; titer ≥ 1E^13^ vg/ml; 5xFAD, *n* = 4; WT, *n* = 4) at bilateral RSC targets (AP, −2.2; ML, ±0.5; DV, 1.0). The gene sequence used to drive ChABC expression has been described by Carstens et al. (2021) based on the plasmid originally generated by Muir and modified by Zhao (Muir et al., 2010; Zhao et al., 2011). Prior to injection, each virus was diluted 1:2 in 0.9% saline. At each target, these injections were delivered in four sets of 50 nl pulses at a rate of 10 nl/s with 10 s between pulses. After all injections were delivered and incisions were closed, mice were returned to standard housing conditions for 1vweek and monitored throughout their recovery. Following the conclusion of the postoperative monitoring period, all mice were transferred to enriched housing conditions (*n* = 8–9 per cage) for 2 months.

### Histology

#### Perfusion and tissue processing

When applicable, mice were deeply anesthetized with isoflurane and transcardially perfused with 0.1 M phosphate-buffered saline (PBS) and 4% formaldehyde 90 min following the completion of the trace conditioning task. In experiments without behavioral testing, mice underwent these same perfusion procedures without any time-locking. In all cases, brains were extracted and postfixed in 4% formaldehyde at 4°C for 24 h. Brains were then submerged in 30% w/v sucrose solution in PBS for 2–3 d until no longer buoyant for cryoprotection. Brains were sagittally sectioned 40 μm thick on a cryostat (Leica CM 1950, Concord) in 12 series. Sections were kept in a buffered antifreeze solution containing 30% ethylene glycol and 20% glycerol in 0.1 M PBS and stored at −20°C.

#### Immunofluorescent staining—mice

For all immunofluorescent staining, free-floating tissue sections were washed three times (10 min per wash) in 0.1 M PBS at room temperature. Sections were then incubated at room temperature in primary antibody solution, containing primary antibody, 3% normal goat serum, and 0.3% Triton X-100. Following primary incubation, the tissue was washed in 0.1 M PBS (3 × 10 min) prior to incubation in secondary antibody solution containing secondary antibody and 0.1 M PBS at room temperature for 24 h.

Parvalbumin staining utilized a 48 h incubation in a 1:5,000 dilution of primary antibody solution (rabbit anti-PV; Invitrogen, PA1–933) followed by a 24 h incubation in a 1:500 dilution of secondary antibody solution (goat anti-rabbit Alexa Fluor 594; Jackson ImmunoResearch Laboratories, 111-585-003 or goat anti-rabbit Alexa Fluor 647; Jackson ImmunoResearch Laboratories, 111-605-003). When staining for c-Fos, sections were incubated in a 1:2,000 dilution of primary antibody solution (rabbit anti-c-Fos; EnCor, RPCA-c-Fos) for 48 h followed by a 24 h incubation in a 1:500 dilution of secondary antibody solution (goat anti-rabbit Alexa Fluor 594; Jackson ImmunoResearch Laboratories, 111-585-003). Synaptotagmin-2 staining involved a 48 h incubation in a 1:200 dilution of primary antibody (mouse anti-ZNP-1; DSHB, F1R5C0) followed by a 24 h incubation in a 1:500 dilution of secondary antibody solution (alpaca anti-mouse Alexa Fluor 594; Jackson ImmunoResearch Laboratories, 615-545-214). Chondroitin-4-sulfate staining utilized a 1:1,000 dilution of primary antibody solution (mouse anti-BE-123; EMD Millipore, MAB2030) for 24 h followed by a 24 h incubation in a 1:500 dilution of secondary antibody (alpaca anti-mouse Alexa Fluor 647; Jackson ImmunoResearch Laboratories, 615-605-214). Amyloid staining was conducted using a dilution of 1:500 primary antibody conjugated to Alexa Fluor 594 (anti-amyloid 17-28 4G8; BioLegend, 800717), and tissues were incubated for 24 h.

PNNs were visualized using WFA staining (Evans et al., 2022). Tissue sections were washed in 0.1 M PBS (3 × 10 min) prior to incubation in carbo-free blocking buffer (VECTOR Labs) with 0.2% Triton X-100 for 30 min. Then, sections were stained in a 1:100 dilution of TRITC-labeled WFA (VECTOR Labs) in carbo-free blocking buffer with 0.05% Tween 20 for 24 h.

Finally, all tissues were counterstained with DAPI (20 min, 1:1,000 in 0.1 M PBS) before being washed in 0.1 M PBS (2 × 10 min) and mounted to glass slides. Slides were coverslipped with PVA-DABCO mounting medium.

#### Immunofluorescent staining—human tissue samples

Upon removal from formalin fixative, human tissue samples were cryoprotected in 30% w/v sucrose solution in 0.1 M PBS for 3–5 d. The tissue was sectioned at a thickness of 50 μm on a cryostat (Leica CM 1950) in 12 series. Tissue series were then stored at −20°C in a buffered antifreeze solution containing 30% ethylene glycol, 20% glycerol, and 0.1 M PBS.

Free-floating human tissue sections were stained as previously described (Epp et al., 2013). Samples were rinsed three times (10 min per wash) in 0.1 M PBS prior to incubation in a primary antibody solution consisting of 3% normal goat serum, 0.3% Triton X-100, a 1:1,000 dilution of rabbit anti-parvalbumin antibody (Invitrogen, PA1-933), and a 1:500 dilution of anti-amyloid (17–28 4G8) antibody conjugated to Alexa Fluor 594 (BioLegend, 800717) for 48 h. Following this incubation, tissue sections were rinsed three more times (10 min per wash) in 0.1 M PBS and incubated for 24 h in a 0.1 M PBS solution containing a 1:500 dilution of alpaca anti-rabbit Alexa Fluor 647 (Jackson ImmunoResearch Laboratories, 611-005-215). Samples were rinsed three more times (10 min per wash) in 0.1 M PBS, mounted to plain glass slides, and then coverslipped using PVA-DABCO mounting medium.

#### TUNEL staining

To assess whether PV-INs are dying in 5xFAD mice, TUNEL staining was conducted. Using 20 μm fresh-frozen, slide-mounted, coronal brain sections from 6-month-old female heterozygous 5xFAD mice and transgene-negative WT littermates, TUNEL staining was conducted as per manufacturer's instructions (Cell Signaling Technology, #48513). Briefly, samples were fixed in 4% formaldehyde for 30 min prior to being washed with 0.1 M PBS (2 × 5 min per wash). Samples were permeabilized during a 30 min incubation in a 0.1 M PBS solution containing 0.2% Triton X-100 prior to more 0.1 M PBS washes (2 × 5 min per wash). Following a 5 min incubation in TUNEL Assay Equilibrium Buffer (Cell Signaling Technology, #84862), samples were stained during a 30 min incubation in a mixture of 2% TdT Enzyme (Cell Signaling Technology, #79533) in CF 594 TUNEL Reaction Buffer (Cell Signaling Technology, #52143). The tissue was washed (3 × 5 min per wash) in 0.2% Triton X-100 and then in 0.1 M PBS (2 × 5 min per wash). Samples were then incubated for 24 in a primary antibody solution containing 3% normal goat serum, 0.3% Triton X-100, and a 1:5,000 dilution of anti-PV antibody (Invitrogen, PA1-933). Following primary incubation, the tissue was washed in 0.1 M PBS (3 × 10 min) prior to incubation in secondary antibody solution containing a 1:500 dilution of alpaca anti-rabbit Alexa Fluor 647 secondary antibody (Jackson ImmunoResearch Laboratories, 611-605-205) and 0.1 M PBS at room temperature for 24 h. Samples were then counterstained with DAPI (20 min, 1:1,000 in 0.1 M PBS) before being washed in 0.1 M PBS (2 × 10 min) and coverslipped with PVA-DABCO mounting medium. Samples were imaged using an OLYMPUS FV3000 confocal microscope equipped with a 40× oil immersion objective (N.A. 1.40).

### Imaging and image analysis

#### Density analyses

The density of parvalbumin, c-Fos, and amyloid expression in the mouse and human brain tissue was assessed using images collected at 10× magnification (N.A. 0.4) using an OLYMPUS VS120-L100-W slide scanning microscope. Labels were segmented from background based on the label size and fluorescent intensity using the user-guided machine learning image processing software *Ilastik*. Binary segmented labels were exported from *Ilastik*, and their expression density or the percentage of total region coverage was assessed within the RSC based on a tracing of this region in the accompanying DAPI channel or autofluorescence image in ImageJ.

The expression density of synaptotagmin-2 across the RSC was assessed using images collected at 60× magnification using an oil immersion objective (N.A. 1.42) equipped to an OLYMPUS FV3000 confocal microscope. Volumetric imaging frames (10 *z* steps; 0.35 μm *z* spacing) were collected from the RSC, and synaptotagmin-2 puncta were segmented via *Ilastik* from maximum intensity projections of these stacks. The density of these labels was assessed using ImageJ.

The optical density of chondroitin 4 sulfate (CS, BE-123, stub labeling) was assessed using images collected at 10× magnification (N.A. 0.4) with an OLYMPUS VS120-L100-W slide scanning microscope. Images were loaded into ImageJ, where the RSC was traced on each section using the DAPI channel as a reference. For each image, the mean pixel intensity of the CS stub labeling was assessed across the RSC.

#### Functional connectivity analyses

Brain-wide c-Fos expression was imaged using an OLYMPUS VA120-L100-W slide scanning microscope equipped with a 10× objective (N.A. 0.4). Using *Ilastik* (Berg et al., 2019), c-Fos^+^ cells were segmented to generate binary masks of brain-wide c-Fos expression. These binary masks were then registered to the Allen Mouse Brain Reference Atlas using a modified protocol building upon *Whole Brain* (Fürth et al., 2018; Terstege et al., 2022). Using custom MATLAB analyses, outputs were organized to yield c-Fos expression densities across 50 neuroanatomical regions (for a detailed list of regions, see Table S1). Regional c-Fos expression density was correlated across groups for all possible combinations of regions, generating correlation matrices of regional coactivation for each group (Wheeler et al., 2013; Vetere et al., 2017; Terstege and Epp, 2022; Ramkumar et al., 2024).

### Statistics and data visualization

All statistical analyses were performed in Prism (GraphPad Software, Version 9.4.0) or with standard MATLAB functions and statistical tests. Independent *t* tests, two-way ANOVAs, and Pearson's correlations were performed. Data in graphs are presented as mean ± SEM. Hypothesis testing was complemented by additional incorporation of estimation statistics for each comparison using estimationstats.com (Ho et al., 2019). These tests provide additional context regarding the magnitude of the observed differences using effect sizes (Cohen's *d*) calculated using a bootstrap sampling distribution with 5,000 resamples along with a 95% confidence interval (CI; bias-corrected and accelerated). Data used in these statistical comparisons were formally determined to be normally distributed using the Shapiro–Wilk test, as such, parametric statistics were applied throughout. Statistical methods were not used to predetermine study sizes but were based on similar experiments previously published [functional connectivity networks, *n* ≥ 7 (Terstege and Epp, 2022; Ramkumar et al., 2024); ChABC histology, *n* ≥ 4 (Bartus et al., 2014; Yang et al., 2015, 2021)]. Experimenters were blinded to the genotype and housing condition of the animals during all analyses. All statistical comparisons and outputs are included in Data S1. All plots were generated in Prism or MATLAB. Circle plots were generated using the circularGraph MATLAB function. The datasets supporting the conclusions of this article have been made available within the Extended Data file uploaded to the Terstege2025A branch of the following GitHub repository: https://doi.org/10.5281/zenodo.17569435.

## Results

### Female 5xFAD mice housed under enriched conditions do not show cognitive impairments

**Figure 1.**
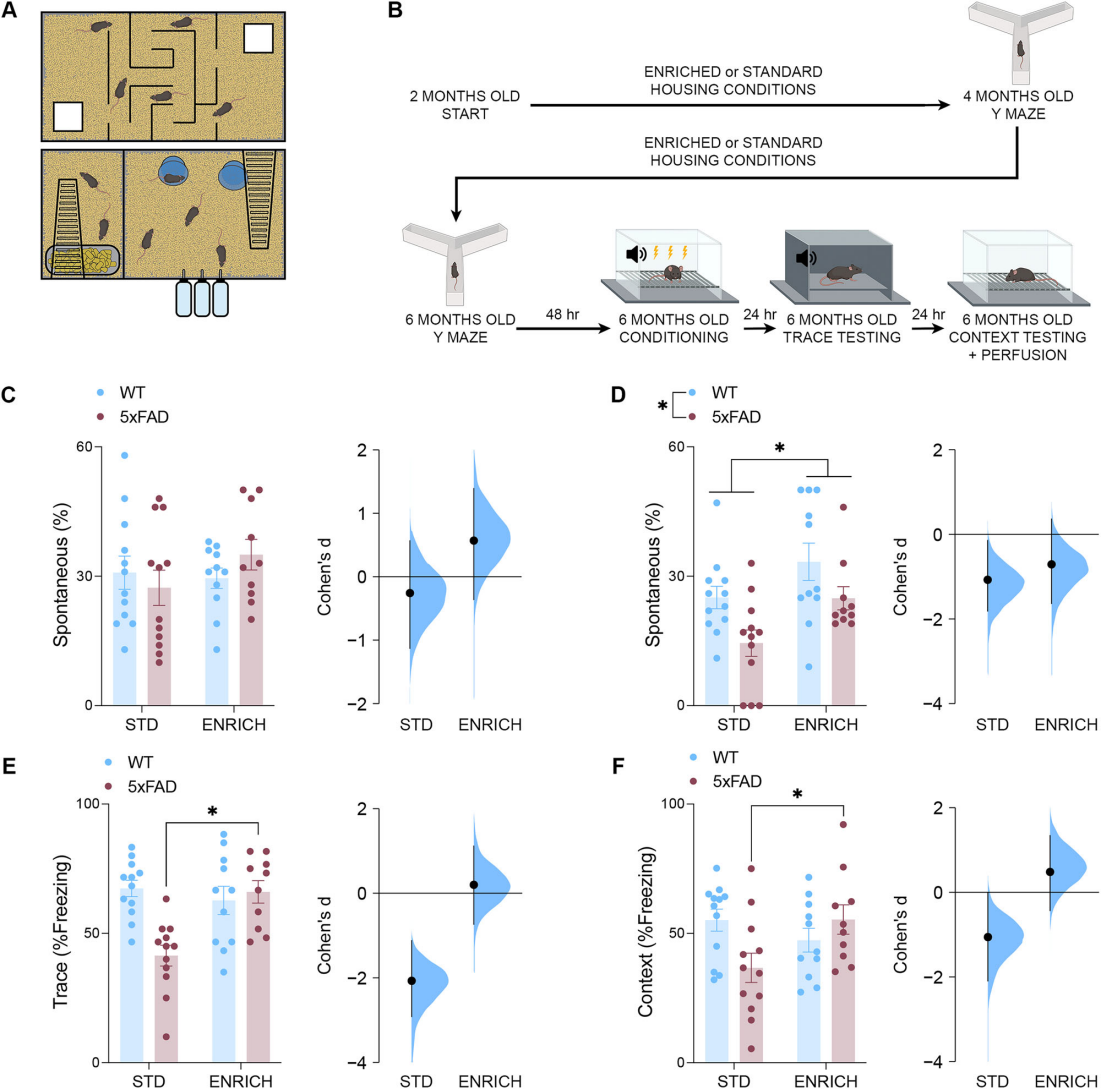
Environmental enrichment protects against cognitive decline. ***A***, Custom-built multilevel cage designed to provide enrichment through cognitive, social, and exercise-related means. ***B***, Timeline of behavioral testing and enrichment. ***C***, The percentage of arm entries in the Y maze which were spontaneous alternations at 4 months old (*n*, WT STD = 12; 5xFAD STD = 12; WT ENRICH = 11; 5xFAD ENRICH = 10). The effect size between WT and 5xFAD mice across control (Cohen's *d* = −0.255) and enriched (Cohen's *d* = 0.203) conditions. ***D***, By 6 months old, 5xFAD mice perform a lower percentage of spontaneous alternations, while mice housed in enriched conditions perform a higher percentage of spontaneous alternations (*n*, WT STD = 12; 5xFAD STD = 12; WT ENRICH = 11; 5xFAD ENRICH = 10, two-way ANOVA, effect of genotype, *F*_(41)_ = 8.606; *p* = 0.0055; effect of housing, *F*_(41)_ = 8.278; *p* = 0.0063). The effect size between WT and 5xFAD mice across control (Cohen's *d* = −1.07) and enriched (Cohen's *d* = −0.71) conditions. ***E***, Six-month-old 5xFAD mice housed under standard conditions spent less time freezing during the trace segment of the testing trial (*n*, WT STD = 12; 5xFAD STD = 12; WT ENRICH = 11; 5xFAD ENRICH = 10, two-way ANOVA, genotype × housing interaction, *F*_(41)_ = 11.65; *p* = 0.0015; Šídák's test, STD 5xFAD vs ENRICH 5xFAD, *p* = 0.0005). The effect size between WT and 5xFAD mice across control (Cohen's *d* = −2.07) and enriched (Cohen's *d* = 0.203) conditions. ***F***, Six-month-old 5xFAD mice housed under standard conditions spent less time freezing upon reintroduction to the conditioned context (*n*, WT STD = 12; 5xFAD STD = 12; WT ENRICH = 11; 5xFAD ENRICH = 10, two-way ANOVA, genotype × housing interaction, *F*_(41)_ = 6.737; *p* = 0.0130; Šídák's test, STD 5xFAD vs ENRICH 5xFAD, *p* = 0.0281). The effect size between WT and 5xFAD mice across control (Cohen's *d* = −1.06) and enriched (Cohen's *d* = 0.482) conditions. Data represent mean ± SEM. *p < 0.05. All statistical comparisons have been provided as Data S1.

The 5xFAD mouse model of AD has a well-characterized timeline of pathological progression and cognitive impairments. At 6 months of age, we observed the expected high density of amyloid-β plaque deposition in the RSC of 5xFAD mice (Fig. S1*A*). Enrichment did not influence the accumulation of amyloid-B plaques compared with standard housing (Fig. S1*B*; see Fig. 1*A* for schematic of enrichment cage). Based on previous characterization of the 5xFAD mice, we also expected that deficits in spatial and conditioned memory should arise by 6 months of age (Terstege et al., 2025; see Fig. 1*B* for timeline of behavioral testing in the current manuscript). Spatial memory performance assessed in the Y maze spontaneous alternation task supported these previous findings. Here, no effects of genotype or housing condition were observed in 4-month-old mice (Fig. 1*C*). However, by 6 months, we observed a lower proportion of spontaneous alternations in 5xFAD mice and an increase in spontaneous alternations in mice housed under enriched conditions (Fig. 1*D*). Subsequent analyses of the effect sizes of these changes by housing condition revealed that the performance of 5xFAD mice was only impaired relative to WT controls when housed under standard conditions.

Following spatial memory testing, mice were trained on a trace contextual fear memory protocol. This protocol allows for the assessment of both cued and contextual memory. In 6-month-old WT and 5xFAD mice, no differences were observed in the rate of freezing behavior during the initial conditioning trial (Fig. S1*C*,*D*). Following tone presentation during a trace testing trial 24 h later, 5xFAD mice housed under control conditions displayed decreased freezing, indicating impaired retrieval of the cued memory (Fig. 1*E*). Subsequent reintroduction to the original conditioned context revealed that these impairments extended to contextual memory performance, with 5xFAD mice housed under standard conditions exhibiting decreased freezing during this trial (Fig. 1*F*). In each of these trials, 5xFAD mice housed under enriched conditions displayed no memory impairments.

### Enriched housing conditions are sufficient to maintain healthy local activity and global functional connectivity of the RSC in female 5xFAD mice

**Figure 2.**
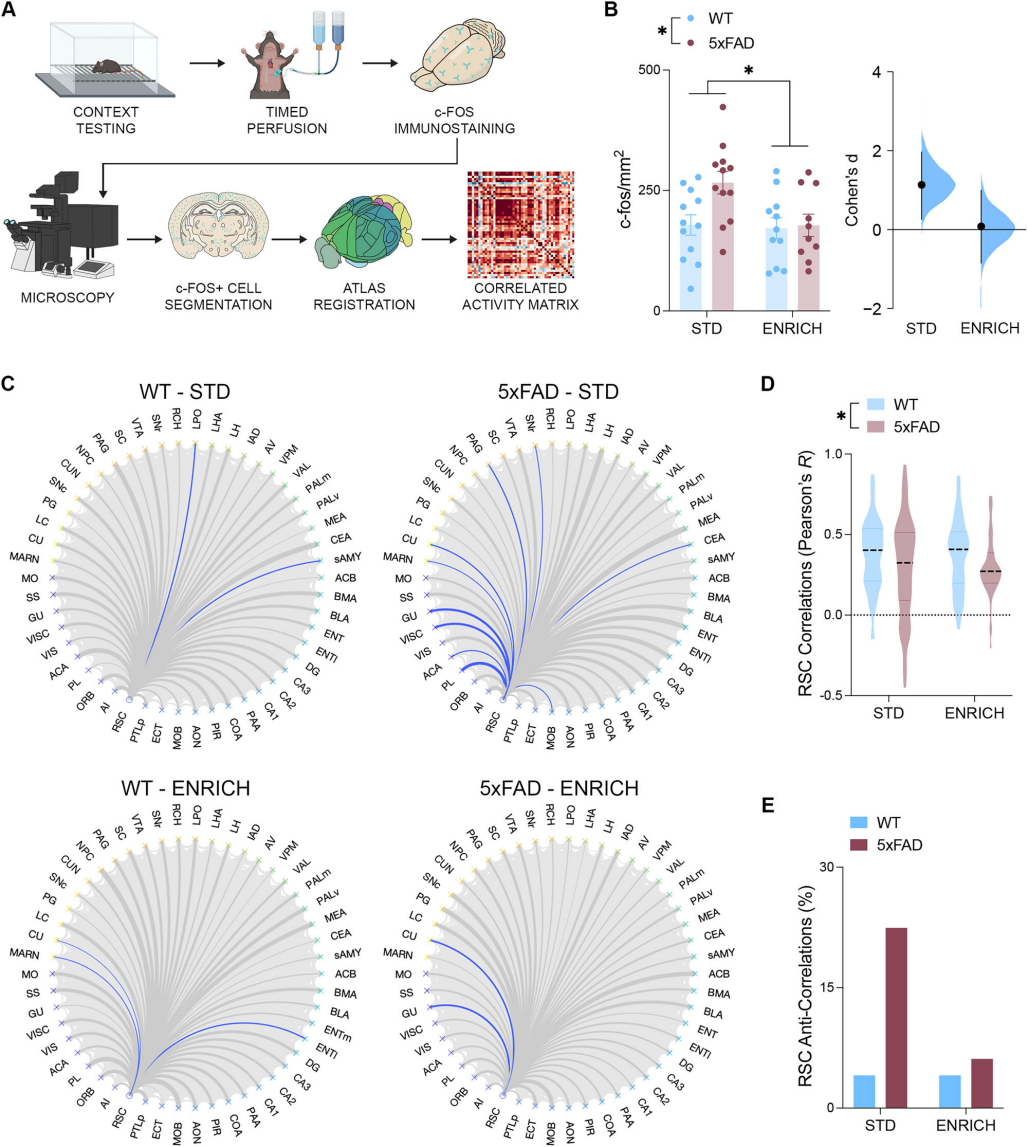
Environmental enrichment protects the functional connectivity of the RSC in 6-month-old 5xFAD mice. ***A***, Schematic outlining the process of generating functional connectivity networks from brain-wide c-fos expression. ***B***, RSC c-fos density was elevated with 5xFAD genotype and standard housing conditions (*n*, WT STD = 12; 5xFAD STD = 12; WT ENRICH = 11; 5xFAD ENRICH = 10, two-way ANOVA, effect of genotype, *F*_(41)_ = 4.352; *p* = 0.0432; effect of housing, *F*_(41)_ = 4.489; *p* = 0.0402). The effect size between WT and 5xFAD mice across control (Cohen's *d* = 1.14) and enriched (Cohen's *d* = 0.084) conditions. ***C***, Circle plots outlining the functional connectome of the RSC. Correlations with positive Pearson's *r* correlation coefficients are depicted in gray, while correlations with negative correlation coefficients (anticorrelations) are depicted in blue. Correlations were derived from the following *n*: WT STD = 12; 5xFAD STD = 12; WT ENRICH = 11; 5xFAD ENRICH = 10. ***D***, Correlations involving the RSC were of a lower mean Pearson's *r* correlation coefficient in 5xFAD mice (two-way ANOVA, effect of genotype, *F*_(192)_ = 5.388; *p* = 0.0219). ***E***, 5xFAD mice housed under standard conditions (STD) had an increased percentage of anticorrelations relative to all other groups (two-proportion *z* test, STD WT vs STD 5xFAD, *z* = −2.6802; *p* = 0.00736; STD 5xFAD vs ENRICH WT, *z* = 2.6802; *p* = 0.00736; STD 5xFAD vs ENRICH 5xFAD, *z* = 2.3094; *p* = 0.2088). Data represent mean ± SEM. *p < 0.05. All statistical comparisons have been provided as Data S1.

To investigate the patterns of brain-wide neuronal activity underlying contextual memory retrieval, 6-month-old mice were perfused 90 min after reintroduction to a contextually conditioned context (see Fig. 2*A* for a schematic outlining these procedures). Subsequent c-fos staining revealed peak neuronal activity during contextual memory retrieval. Within the RSC, c-fos expression density was elevated with both standard housing conditions and 5xFAD genotype (Fig. 2*B*). The effect size of this RSC hyperactivity was greatest in 5xFAD mice housed under standard conditions.

To determine the impact of RSC hyperexcitability on the ability of this region to communicate effectively with other brain regions during the retrieval of a conditioned memory, c-fos expression density was assessed across 49 other brain regions and correlated with that of the RSC. The resulting correlations outlined the nature of the patterns of coactivity between the RSC and the rest of the network (Fig. 2*C*,*D*). In 6-month-old mice, the mean magnitude of the correlation coefficient between the RSC and the other regions of the network was decreased with 5xFAD status (Fig. 2*E*). However, the number of correlations with a Pearson's *R* of a negative magnitude (anticorrelations) involving the RSC was only elevated above control levels in the 5xFAD mice housed under control conditions, with enriched housing conditions attenuating this effect (Fig. 2*F*).

### Density and synaptic connectivity of PV-INs in the RSC are preserved in female 5xFAD mice under enriched housing conditions

**Figure 3.**
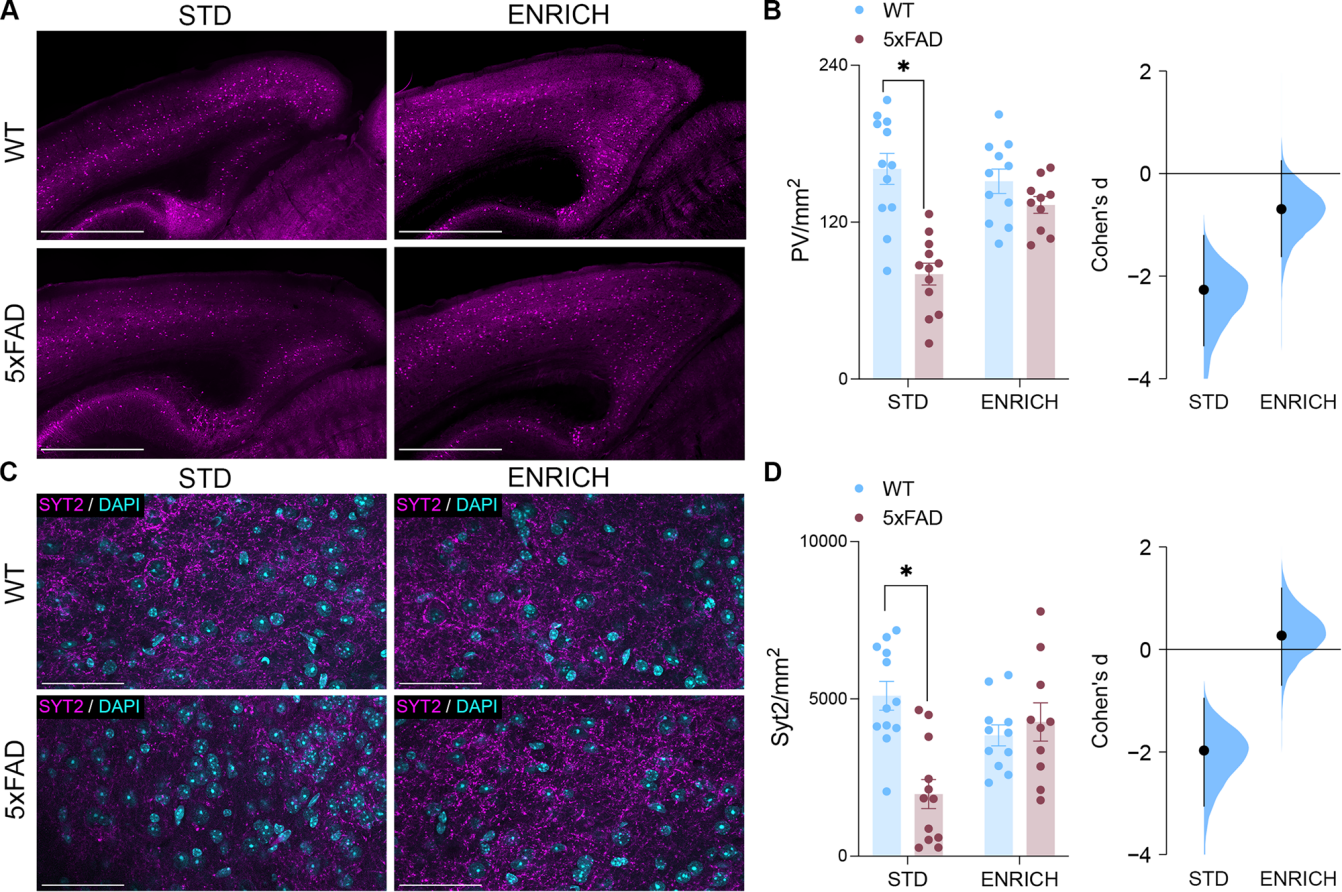
PV-IN density and connectivity are preserved with environmental enrichment. ***A***, PV staining across the RSC. Scale bars represent 1,000 μm. ***B***, RSC PV density was decreased in 6-month-old 5xFAD mice housed under standard conditions (*n*, WT STD = 12; 5xFAD STD = 12; WT ENRICH = 11; 5xFAD ENRICH = 10, two-way ANOVA, genotype × housing interaction, *F*_(41)_ = 10.83; *p* = 0.0021; Šídák's test, STD WT vs STD 5xFAD, *p* < 0.0001). The effect size between WT and 5xFAD mice across control (Cohen's *d* = −2.26) and enriched (Cohen's *d* = −0.689) conditions. ***C***, SYT2 staining across the RSC. Scale bars, 50 μm. ***D***, RSC SYT2 puncta density was decreased in 6-month-old 5xFAD mice housed under standard conditions (*n*, WT STD = 12; 5xFAD STD = 12; WT ENRICH = 11; 5xFAD ENRICH = 10, two-way ANOVA, genotype × housing interaction, *F*_(41)_ = 14.29; *p* = 0.0005; Šídák's test, STD WT vs STD 5xFAD, *p* < 0.0001). The effect size between WT and 5xFAD mice across control (Cohen's *d* = −1.97) and enriched (Cohen's *d* = 0.275) conditions. Data represent mean ± SEM. *p < 0.05. All statistical comparisons have been provided as Data S1.

It has previously been proposed that anticorrelated functional connectivity may be influenced in part by the activity of PV-INs (Mizuseki and Buzsaki, 2014; Moretti et al., 2022; Terstege et al., 2025). Furthermore, it has also previously been established that PV-IN density is vulnerable in 5xFAD mice (Park et al., 2024; Terstege et al., 2025). To assess whether the preserved functional connectivity of the RSC in 5xFAD mice housed under enriched conditions was mediated in part through PV-IN, brains of 6-month-old mice were labeled for this protein (Fig. 3*A*). A significant genotype × housing condition interaction revealed a reduction in PV-IN expression density across the RSC of 5xFAD mice housed under standard conditions. Furthermore, a TUNEL assay with concurrent PV staining revealed PV-INs undergoing programmed cell death (Fig. S1*E*). The presence of programmed cell death coincides with previous findings showing decreased densities of PV-INs and NeuN^+^ neurons in the RSC of 5xFAD mice compared with age-matched WT mice (Terstege et al., 2025). However, this deficit in PV-IN expression density was not present in 5xFAD mice housed under enriched conditions (Fig. 3*B*).

To assess the functional consequences of this decrease in PV-IN expression density across the RSC, we stained the tissues from 6-month-old mice for synaptotagmin-2 (SYT2; Fig. 3*C*). SYT2 is a marker with high specificity for presynaptic contacts from PV-INs; therefore, its expression density provides insight into the ability of PV-INs to regulate both its own activity and the activity of pother cell populations across the region (Sommeijer and Levelt, 2012). Here, a significant genotype × housing condition interaction revealed impaired expression density of SYT2 across the RSC of only the 5xFAD mice which were housed under control conditions (Fig. 3*D*).

### Enriched housing conditions are sufficient to preserve the density of perineuronal nets in the RSC of 6-month-old female 5xFAD mice

**Figure 4.**
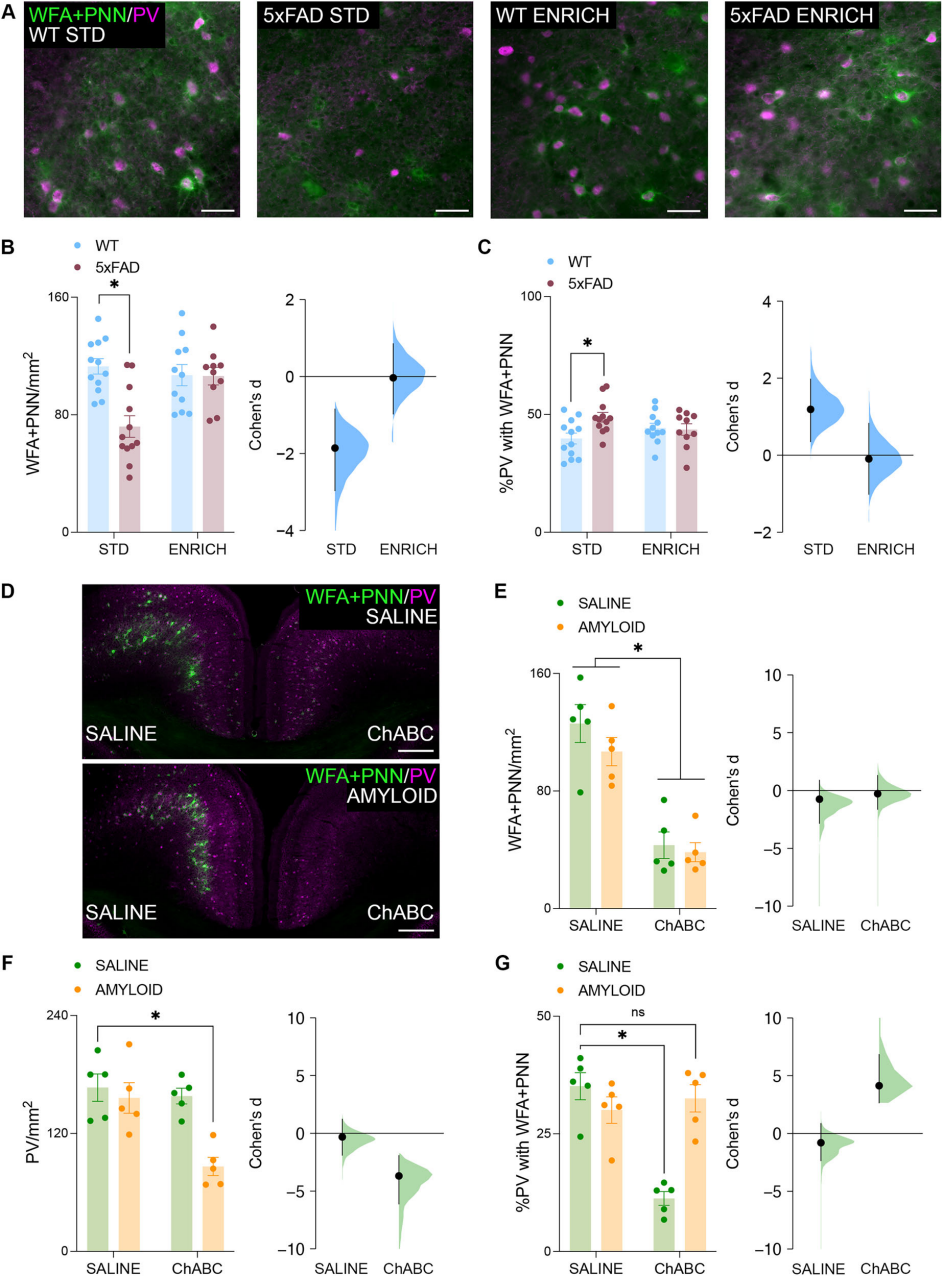
WFA^+^ PNN density is preserved in 6-month-old 5xFAD mice with enrichment and their expression protects PV-INs from amyloid toxicity. ***A***, WFA staining for PNNs across the RSC. Scale bars, 50 μm. ***B***, RSC WFA^+^ PNN density was decreased in 5xFAD mice housed under standard conditions (*n*, WT STD = 12; 5xFAD STD = 12; WT ENRICH = 11; 5xFAD ENRICH = 10, two-way ANOVA, genotype × housing interaction, *F*_(41)_ = 9.498; *p* = 0.0037; Šídák's test, STD WT vs STD 5xFAD, *p* < 0.0001). The effect size between WT and 5xFAD mice across control (Cohen's *d* = −1.86) and enriched (Cohen's *d* = −0.0301) conditions. ***C***, The percentage of PV cells in the RSC surrounded by WFA^+^ PNNs was increased in 5xFAD mice housed under standard conditions (*n*, WT STD = 12; 5xFAD STD = 12; WT ENRICH = 11; 5xFAD ENRICH = 10, two-way ANOVA, genotype × housing interaction, *F*_(41)_ = 4.836; *p* = 0.0336; Šídák's test, STD WT vs STD 5xFAD, *p* = 0.0091). The effect size between WT and 5xFAD mice across control (Cohen's *d* = 1.19) and enriched (Cohen's *d* = −0.0927) conditions. ***D***, WFA and PV staining across the RSC of 9-week-old C57BL/6J mice following bilateral saline (top) or amyloid (bottom) infusion at 8 weeks old. All mice were also infused with saline (left hemisphere) or ChABC (right hemisphere). Scale bars, 250 μm. ***E***, ChABC administration reduced the density of WFA^+^ PNNs in the ipsilateral hemisphere of 9-week-old C57BL/6J mice relative to the contralateral hemisphere (*n*, saline–saline = 5; saline–ChABC = 5; amyloid–saline = 5; amyloid–ChABC = 5, two-way ANOVA, effect of ChABC, *F*_(16)_ = 60.06; *p* < 0.0001). The effect size between saline- and amyloid-infused mice across control (Cohen's *d* = −0.752) and ChABC-infused (Cohen's *d* = −0.271) hemispheres. ***F***, ChABC and amyloid coadministration reduced RSC PV density in 9-week-old C57BL/6J mice (*n*: saline–saline = 5; saline–ChABC = 5; amyloid–saline = 5; amyloid–ChABC = 5; two-way ANOVA, amyloid × ChABC interaction, *F*_(16)_ = 6.368; *p* = 0.0226; Šídák's test, saline–saline vs amyloid–ChABC, *p* = 0.0007). The effect size between saline- and amyloid-infused mice across control (Cohen's *d* = −0.319) and ChABC-infused (Cohen's *d* = −3.69) hemispheres. ***G***, ChABC administration reduces the percentage of RSC PV surrounded by WFA^+^ PNNs, but coadministration of ChABC and amyloid does not in 9-week-old C57BL/6J mice (*n*, saline–saline = 5; saline–ChABC = 5; amyloid–saline = 5; amyloid–ChABC = 5; two-way ANOVA, amyloid × ChABC interaction, *F*_(16)_ = 25.89; *p* = 0.0001; Šídák's test, saline–saline vs saline–ChABC, *p* < 0.0001; saline–saline vs amyloid–ChABC, *p* = 0.4879). The effect size between saline- and amyloid-infused mice across control (Cohen's *d* = −0.798) and ChABC-infused (Cohen's *d* = 4.14) hemispheres. Data represent mean ± SEM. *p < 0.05. All statistical comparisons have been provided as Data S1.

One factor that has been implicated in the survival of PV-INs is the presence of WFA^+^ PNNs. In the cortex, these extracellular matrix structures envelope primarily PV-INs and play important roles in regulating their plasticity while also protecting against oxidative stress. As such, compromised WFA^+^ PNN populations may contribute to vulnerability in PV-INs. WFA staining and PV labeling were performed across the RSC (Fig. 4*A*). Histological analyses revealed a significant genotype × housing condition interaction, wherein 5xFAD mice housed under standard conditions displayed impaired expression density of WFA^+^ PNNs across the RSC (Fig. 4*B*). Further histological analyses revealed that, of the PV-INs which remained in the RSC of 5xFAD mice housed under standard conditions, a higher percentage of PV-INs were enveloped by WFA^+^ PNNs (Fig. 4*C*). This result alludes to a potentially protective role of WFA^+^ PNNs on PV-IN populations in AD neuropathology.

### The presence of WFA^+^ PNNs improves the resilience of PV-IN expression to amyloid-β toxicity

In the prior experiment we observed potential protective effect of WFA^+^ PNNs on the expression of PV-INs in 5xFAD mice. To further investigate this relationship, we used targeted infusions of amyloid-β, ChABC, or the coadministration of both in 8-week-old C57BL/6J mice. At 9 weeks old, mice were perfused for histological analyses. As expected, the administration of ChABC reduced the density of WFA^+^ PNNs across the RSC of mice perfused 7 d after surgery (Fig. 4*D*). This coincided with an increase in the optical density of CS stubs across the RSC (Fig. S2*A*,*B*). There was also a significant main effect of amyloid-β on WFA^+^ PNN density; however, the magnitude of this effect size was considerably lower than that of ChABC. There was no significant amyloid- β × ChABC interaction effect on the density of WFA^+^ PNNs. Conversely, when examining the density of PV-INs, we observed a significant amyloid-β × ChABC interaction (Fig. 4*E*). Šídák post hoc comparisons further revealed that the density of PV-INs across the RSC was not directly influenced by either treatment on its own but became significantly impaired only when ChABC and amyloid-β were administered together.

Looking more closely at the interaction between surviving PV-INs and WFA^+^ PNNs under these conditions, we found that without amyloid-β administration, infusion of ChABC alone caused a reduction in the proportion of PV-INs surrounded by WFA^+^ PNNs (Fig. 4*F*). However, with ChABC and amyloid-β coinfusion, this proportion no longer differs from controls (Fig. 4*G*). With the injection of heat-inactivated ChABC (HI-ChABC; Fig. S2*C*), neither the density of WFA^+^ PNNs (Fig. S2*D*), density of PV-INs (Fig. S2*E*), or the percentage of PV-INs enrobed by WFA^+^ PNNs (Fig. S2*F*) across the RSC differed from saline-injected controls.

### The density of RSC PV-INs in the clinical AD cases is anticorrelated with the amyloid burden in this region

**Figure 5.**
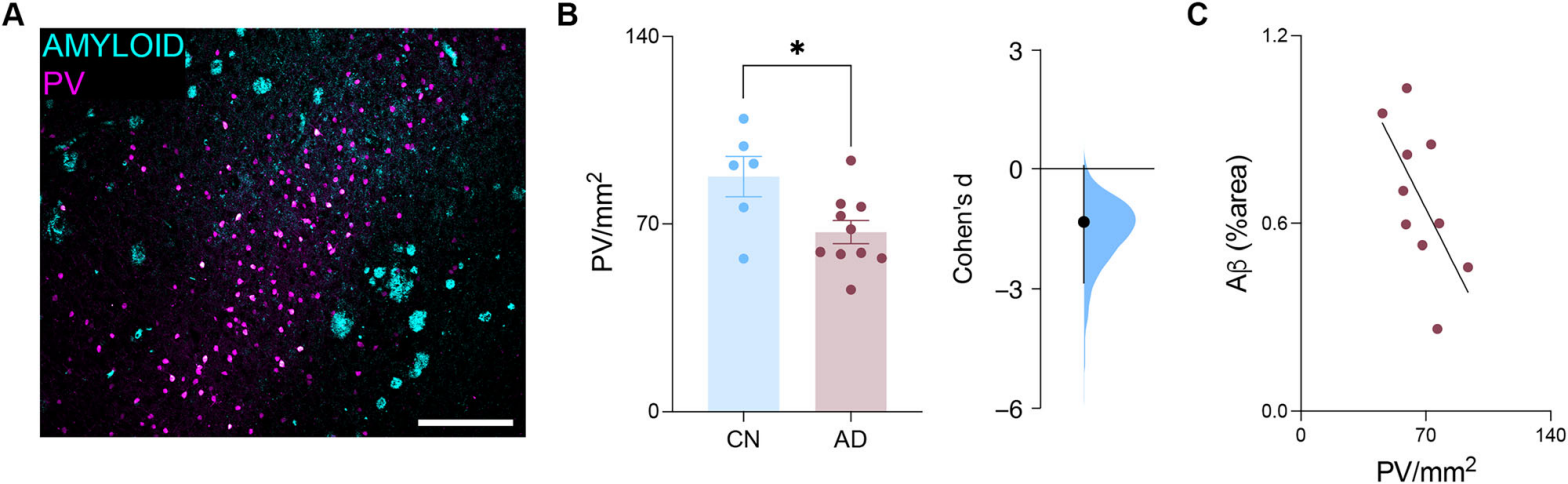
RSC PV-IN density is influenced by amyloid-β load in clinical AD cases. ***A***, Representative photomicrograph illustrating PV and amyloid-β labeling across the RSC. Scale bars, 250 μm. ***B***, RSC PV-IN density was decreased in women with AD (*n*, CN = 6; ENRICH = 10; two-sample *t* test, *t*_(14)_ = 2.585; *p* = 0.0.0216). The effect size between CN and AD tissue samples (Cohen's *d* = −1.33). ***C***, PV-IN density was anticorrelated with the percent coverage of amyloid-β labeling across the RSC of AD patients (Pearson's *r* = −0.6472; *p* = 0.0431; simple linear regression, *y* = −0.01123*x* + 1.433). Data represent mean ± SEM. *p < 0.05. All statistical comparisons have been provided as Data S1.

The relationship between increased amyloid-β load and RSC PV-IN density can also be studied in clinical AD populations through histological analyses of postmortem tissue samples (Fig. 5*A*). By labeling PV-INs across postmortem tissue samples from the RSC, we found that the density of these cells is decreased in women with AD compared with CN controls (Fig. 5*B*). Furthermore, the density of RSC PV-INs in tissue samples obtained from women with AD was anticorrelated with the coverage of amyloid-β burden, wherein higher amyloid-β load was associated with a decreased density of RSC PV-INs (Fig. 5*C*).

### The reduction of WFA^+^ PNNs in the RSC counteracts the protective effects of enrichment on the density of PV-INs in 6-month-old female 5xFAD mice

**Figure 6.**
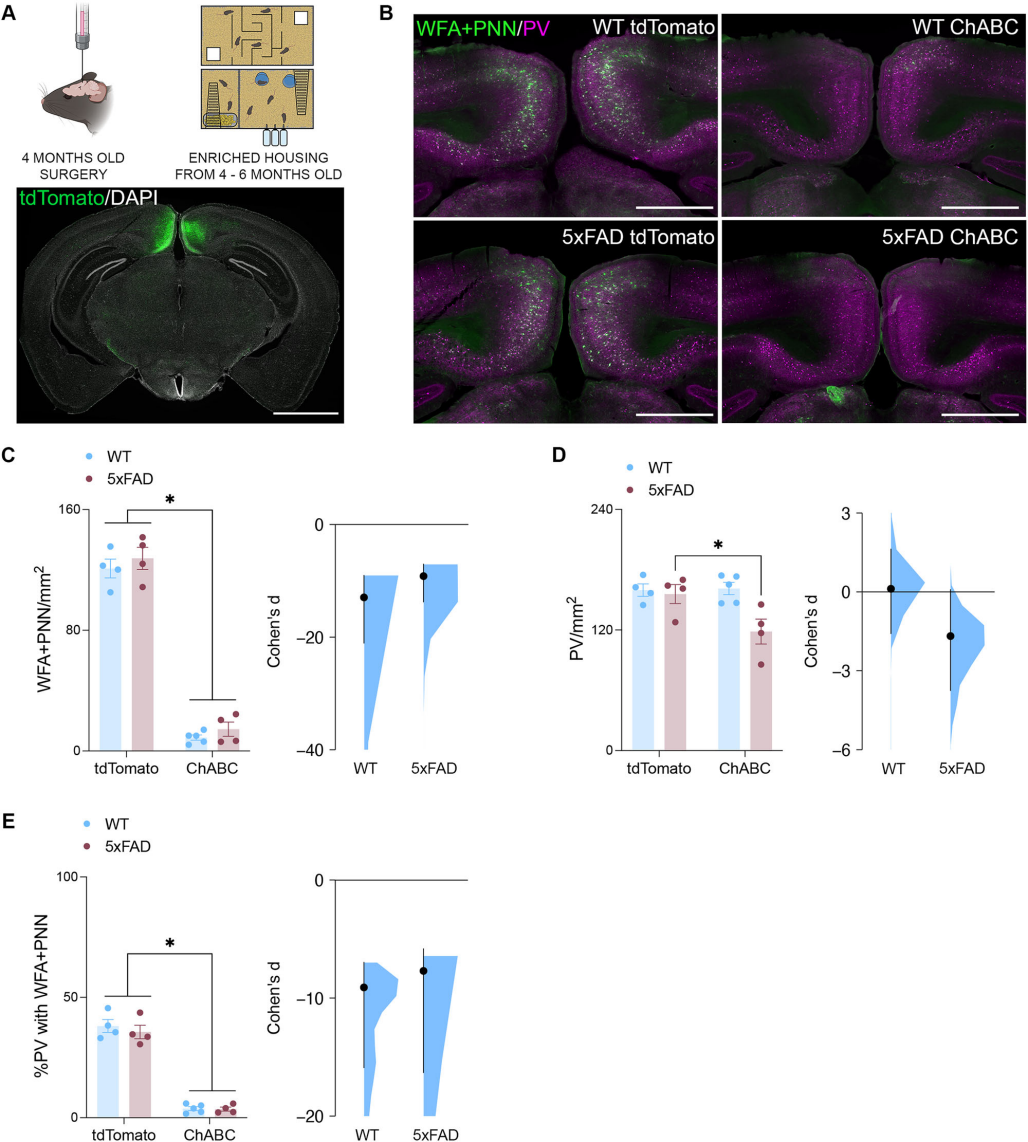
Chronic WFA^+^ PNN depletion suppresses the protective effects of enrichment on PV-IN density in 6-month-old 5xFAD mice. ***A***, Schematic outlining timeline of surgical procedures and enriched housing. Representative photomicrograph illustrating the viral labeling within the RSC. Scale bars, 1,000 μm. ***B***, WFA staining for PNNs across the RSC. Scale bars, 500 μm. ***C***, ChABC expression reduced the density of WFA^+^ PNNs in the RSC (*n*, WT-tdTomato = 4; WT-ChABC = 5; 5xFAD-tdTomato = 4; 5xFAD-ChABC = 4, two-way ANOVA, effect of ChABC, *F*_(13)_ = 483.0; *p* < 0.0001). The effect size between saline- and amyloid-infused mice across WT (Cohen's *d* = −12.9) and 5xFAD (Cohen's *d* = −9.18) mice. ***D***, ChABC expression reduced RSC PV density in 5xFAD mice (*n*, WT-tdTomato = 4; WT-ChABC = 5; 5xFAD-tdTomato = 4; 5xFAD-ChABC = 4, two-way ANOVA, genotype × ChABC interaction, *F*_(13)_ = 4.978; *p* = 0.0439; Šídák's test, 5xFAD-tdTomato vs 5xFAD-ChABC, *p* = 0.0224). The effect size between saline- and amyloid-infused mice across WT (Cohen's *d* = 0.807) and 5xFAD (Cohen's *d* = −1.68) mice. ***E***, ChABC expression reduced the percentage of PV surrounded by WFA^+^ PNNs in the RSC (*n*, WT-tdTomato = 4; WT-ChABC = 5; 5xFAD-tdTomato = 4; 5xFAD-ChABC = 4; two-way ANOVA, effect of ChABC, *F*_(13)_ = 296.1; *p* < 0.0001). The effect size between saline- and amyloid-infused mice across WT (Cohen's *d* = −9.1) and 5xFAD (Cohen's *d* = −7.7) mice. Data represent mean ± SEM. *p < 0.05. All statistical comparisons have been provided as Data S1.

With the presence of WFA^+^ PNNs improving the resilience of PV-INs to amyloid toxicity, we next asked whether this relationship was critical for mediating the protective effect of enrichment on RSC PV-IN expression in female 5xFAD mice. To investigate this further, we used viral vectors to promote the long-term expression of ChABC or a control virus in RSC PV-INs of female WT and 5xFAD mice housed under enriched conditions (Fig. 6*A*,*B*). Across both WT and 5xFAD mice, the viral expression of ChABC decreased the density of WFA^+^ PNNs (Fig. 6*C*) and increased the optical density of CS stubs across the RSC (Fig. S3*B*). Concerning PV-INs, a significant genotype × virus interaction was observed, wherein decreased expression of WFA^+^ PNNs had no impact on the density of these cells across the RSC of WT mice. However, in 5xFAD mice, Šídák post hoc comparisons further revealed that the viral expression of ChABC and subsequent decreased in WFA^+^ PNNs coincided with decreased expression density of PV-INs across the RSC (Fig. 6*D*). The viral expression of ChABC also decreased the percentage of PV-INs across the RSC which were enrobed by WFA^+^ PNNs (Fig. 6*E*).

## Discussion

Previous studies have identified relationships between enrichment and increased resiliency to cognitive decline in AD (Oveisgharan et al., 2020; Simone et al., 2023). However, there is little consensus as to the mechanism underlying this effect. As the incidence of AD rises, it is critical to gain a better understanding of these accessible lifestyle interventions which could be integrated into AD prevention strategies.

While the mechanisms mediating the protective effects of enrichment on cognitive decline in AD are largely unknown, several neurological correlates predictive of AD onset have been identified (Dubois et al., 2023; Hansson et al., 2023; Jia et al., 2024). Altered RSC activity may predict the onset of cognitive impairment in AD (Ash et al., 2016; Ziontz et al., 2021; Walsh et al., 2022; Veréb et al., 2023). The RSC is important for learning and memory, and its activity can be modulated through learning, social interactions, and physical exercise (Vann et al., 2000; Holschneider et al., 2007; Holzschneider et al., 2012; Todd et al., 2015; Pan et al., 2022; Shi et al., 2024). Therefore, the core elements of our enrichment protocol—cognitive, social, and physical enrichment—all influence the activity of this region. The current study examined whether multimodal enrichment could improve cognitive outcomes and preserve RSC function in the 5xFAD mouse model of AD. With enrichment beginning early, prior to the establishment of significant RSC amyloid pathology and the onset of cognitive impairments, the study mimicked a preventative approach.

After enrichment, 5xFAD mice showed improved spatial working memory and contextual memory compared with 5xFAD mice in standard housing. 5xFAD mice in enriched housing also did not display RSC hyperactivity during contextual memory recall like the 5xFAD mice housed under standard conditions. Furthermore, the density of anticorrelated functional connections formed with the RSC during contextual memory recall was low in 5xFAD mice housed under enriched conditions. This mimicked the density of these connections in WT mice and sharply contrasted the high density of anticorrelated connections observed in 5xFAD mice housed under standard conditions. These changes in RSC functional connectivity in standard housed 5xFAD mice aligned with previous studies (Terstege et al., 2025). Anticorrelated activity involving this region has been reported during early stages of AD, while other reports have shown decreases in RSC anticorrelated activity with physical exercise (Voss et al., 2016; Wang et al., 2019). Here, we show that in AD, the protective effects of enrichment sufficiently suppress RSC dysfunction on a functional connectivity network level.

Recent literature has demonstrated increased vulnerability of PV-INs during the early stages of cognitive decline (Satoh et al., 1991; Solodkin et al., 1996; Hijazi et al., 2023; Terstege and Epp, 2023). PV-INs have also been thought to contribute to anticorrelated functional connectivity profiles (Mizuseki and Buzsaki, 2014; Moretti et al., 2022). During the early stages of cognitive decline, RSC hypometabolism puts considerable strain on metabolically expensive PV-INs (Wree et al., 1993; Nestor et al., 2003a; Povysheva et al., 2019; Terstege et al., 2024). In standard housed 5xFAD mice, we found a decrease in the density of PV-INs and PV-IN presynaptic contacts across the RSC. These impairments were not present in 5xFAD mice in enriched conditions. It is challenging to determine the extent to which the reduction in PV-IN density downregulation of the parvalbumin protein versus cell death of the eponymous PV-INs. We have observed TUNEL^+^ PV-INs in the RSC showing evidence that at least some PV-INs may be dying; this approach does not allow for quantification of how many PV-INs have died. We have addressed this issue in a previous paper where we indelibly labeled PV-INs using a viral approach and later looked for colocalization of parvalbumin using IHC. In doing so, we found that there was a 13% reduction in the number of PV-INs expressing parvalbumin but a 40% reduction in the number of PV-INs (Terstege et al., 2025). This suggests that while most missing RSC PV-INs are dying, there is a subgroup that are still present but lacking parvalbumin expression.

PNNs are involved in both protecting PV-INs and regulating their synaptic connectivity. In 5xFAD mice housed under standard conditions, the density of WFA^+^ PNNs was reduced across the RSC. Enriched housing was sufficient to maintain normal WFA^+^ PNN expression. PNN depletion has been reported in mouse models of AD and tissue samples collected from human subjects with AD (Kobayashi et al., 1989; Seeger et al., 1996; Crapser et al., 2020). Furthermore, environmental enrichment is sufficient to preserve PNN density in mouse models of AD (Cattaud et al., 2018). We extended upon these findings by demonstrating that remaining PNNs are more likely to surround PV-INs than other cell types, suggesting that PV-INs themselves may also serve a protective role in the maintenance of PNNs in the RSC of AD models.

To further examine the protective effects of WFA^+^ PNNs against amyloid infusion, RSC PNNs were directly manipulated using ChABC. No loss of PV-INs occurred in response to amyloid infusion, unless WFA^+^ PNNs had been depleted beforehand. Interestingly, the percentage of PV-INs enrobed by WFA^+^ PNNs decreased with ChABC treatment; however, combined amyloid and ChABC treatments did not alter this percentage. These results suggest that surviving PV-INs were those that maintained their WFA^+^ PNN expression, further demonstrating the protective role of these structures.

To elucidate the relationship between enrichment, PNNs, and PV-IN resilience to AD pathology, ChABC was expressed across RSC PV-INs of WT and 5xFAD mice housed under enriched conditions. Without RSC PNNs, the protective effect of enriched housing conditions on RSC PV-IN density in 5xFAD mice was lost. These findings point toward PNN stability as a potential mechanism mediating the protective effects of enrichment on RSC PV-INs.

Throughout these experiments, PNNs were visualized with WFA staining. In many regions, this method does not exhaustively label all PNNs. WFA staining binds the *N*-acetylgalactosamine moiety of chondroitin sulfate glycosaminoglycans, a critical constituent of PNNs, and is the most commonly used marker to visualize these structures (Härtig et al., 1999; Fawcett et al., 2019; Lupori et al., 2023). Additionally, PNNs are also defined by an aggrecan core, which can be visualized with ACAN staining (Giamanco et al., 2010; Ueno et al., 2018). In the current study, the core ACAN component of PNNs was not investigated. In WFA-labeled PNNs, while *Acan* gene expression often correlates highly with WFA diffuse fluorescence, the extent to which these immunofluorescent labels colocalize is highly variable (Yamada and Jinno, 2017; Ueno et al., 2018; Lupori et al., 2023). In some regions, such as CA3 of the hippocampus, many PNNs label positively for ACAN but do not stain with WFA (Härtig et al., 2022). In the RSC, however, many more PNNs are WFA^+^ than not (Härtig et al., 2022). As such, we focused on WFA^+^ PNNs.

The current study was conducted in female mice. Sex differences are prominent in AD, with women having an increased risk of developing AD (Gao et al., 1998; Andersen et al., 1999). Furthermore, PV-INs are vulnerable in the early stages of AD pathogenesis and highly sensitive to changes occurring during aging in women (Povysheva et al., 2019; Gilfarb and Leuner, 2022). Previous findings identified PV-INs as being particularly susceptible to AD pathogenesis in females, particularly in the RSC where sex-specific impairments of PV-INs occur earlier than in many other neuroanatomical regions commonly associated with AD (Terstege and Epp, 2023; Terstege et al., 2025). As such, the current study was focused on environmental enrichment and its role in mediating this sex-specific vulnerability.

The current study highlights the relationship between enrichment and the proportion of WFA^+^ PNNs enrobing PV-INs in AD; however, they have implications which could shed light on a multitude of neuroscience research topics. In the hippocampus, alterations in PNN and PV-IN colocalization have been shown to impact the ability to form and maintain memories (Liu et al., 2023; Ramsaran et al., 2023). In the entorhinal cortex, impaired coverage of PNNs around PV-INs has been associated with the development of epilepsy (Rogers et al., 2018; Yang et al., 2022). While these relationships have been well characterized, there have been relatively few studies identifying means through which the density of WFA^+^ PNNs can be restored or protected. The current study demonstrates this protective effect and has the potential to inspire new avenues of research in other neuroscience fields.

While many studies have reported associations between cognitive, social, and physical enrichment on resilience to cognitive decline in AD; several studies have failed to replicate these results (Jankowsky et al., 2005; Hüttenrauch et al., 2017; Butler et al., 2018; Griñán-Ferré et al., 2018; Oveisgharan et al., 2020). In our results, it is important to highlight potential confounds which may contribute to variability in the literature. One key contributor may be protocol duration (Kallio et al., 2017; Vasquez et al., 2022; Wang et al., 2022). Generally, shorter enrichment protocols have yielded smaller effects (Hampshire et al., 2019). Moreover, the variety of enrichment is important (Kelly et al., 2014), and early intervention is most effective in slowing cognitive decline. Here, we have implemented a long multimodal enrichment protocol prior to the onset of cognitive symptoms to maximize our effects.

## Conclusion

Our findings demonstrate that prolonged cognitive, social, and physical enrichment abolishes cognitive deficits observed in 6-month-old female 5xFAD mice. Our results also suggest that these conditions are sufficient to preserve functional connectivity of the RSC and protect PV-INs in this region, even in the absence of differences in amyloid pathology. Together, these results provide support for the maintenance of PV-INs in the RSC as a potential mechanism mediating the protective effects of enrichment against cognitive decline in AD.
